# Disposal practice and factors associated with unused medicines in Malaysia: a cross-sectional study

**DOI:** 10.1186/s12889-021-11676-x

**Published:** 2021-09-16

**Authors:** Leong Seng Wang, Zoriah Aziz, Zamri Chik

**Affiliations:** 1 Faculty of Medicine, University of Malaya, 50603 Kuala Lumpur, Malaysia; 2 School of Pharmacy, Monash University, 47500 Bandar Sunway, Selangor Malaysia; 3 Faculty of Pharmacy, MAHSA University, 42610 Jenjarom, Selangor Malaysia

**Keywords:** Disposal, Unused medicines, Logistic regression, Predictors, Chronic illnesses, Acute illnesses

## Abstract

**Background:**

The improper disposal of unused medicines is a worldwide concern because of its impact on the environment, economy, and health. This study aims to describe the disposal practice of unused medicine and identify factors associated with unused medicines in Malaysia.

**Methods:**

This was a cross-sectional, face to face interview-based survey using a structured questionnaire. We used a convenience sampling method to recruit participants from Kuala Lumpur and Selangor in Malaysia.

**Results:**

We interviewed 1184 participants, and the response rate was 96%. Out of the total respondents, 995 (84%) reported having unused medicines. About a quarter of respondents kept unused medicines in the cabinet, and another quarter disposed of them into the trash or toilet. Only half of the respondent who used medicines for chronic illnesses had unused medicines compared to about 90% of respondents who used medicines for acute illnesses. The main reason for having unused medicines among those who used medicines for chronic illness was non-adherence (69%, *p* <  0.05). Only 27% of these respondents returned unused medicines under the “Medicine Return Programme (MRP)”. The other group who used medicines for acute illnesses had unused medicines because their health conditions improved. Thus, most of the unused medicines will eventually end up in household waste. A multivariate logistic regression analysis identified respondents who used medicines for acute illnesses as the strongest predictor of having unused medicines (Odds Ratio (OR) = 29.8; *p* <  0.001), followed by those who pay for their medicines (OR = 6.0; *p* < 0.001) and those who were willing to participate the Medicine Return Programme (OR = 2.5; *p* = 0.009).

**Conclusion:**

The prevalence of unused medicines and their improper disposal were high in Malaysia. Unused medicines are associated with people who use medicines for acute illnesses, pay for their medication, and are willing to participate in an MRP. Rationale prescription and optimal dispensing practice, together with a broader MRP facilities coverage, could reduce unused medicine possession.

**Supplementary Information:**

The online version contains supplementary material available at 10.1186/s12889-021-11676-x.

## Background

Unused medicines are defined as pharmaceuticals that are no longer consumed by the intended users or patients [1]. Patients could possess unused medicines for a number of reasons, including non-adherence to their treatment, improvement of their medical condition, change in their treatment, experienced adverse effects and oversupplied with medicines from multiple centres [1–4]. These unused medicines could be improperly disposed of. For the context of this paper, improper disposal of unused medicines is defined as discarding unused medicine other than returning them to a Medicine Return Programme (MRP). Under the programme, the collected unused medicine will be disposed of as pharmaceutical waste through regulated incineration [5–9].

Unused medicines improperly disposed of, for example, medicines discarded in the household garbage, will end up in the landfill and subsequently contaminate surface water. Unused medicines may also be flushed into the toilet or drain that are channelled into the sewage system, resulting in direct surface water contamination [4, 8]. The contaminated surface water with pharmaceutical ingredients would harm humans, animals and aquatic lives [4]. The management of these pharmaceutical ingredients in the environment is both challenging and potentially costly [10]. Unused medicines not only cause harmful effects on the environment, their improper storage and disposal might also lead to misuse by unintended users and causing accidental childhood poisoning [11, 12].

As the improper disposal of unused medicines causes significant concern worldwide, including in Malaysia [3, 12–19], the Ministry of Health, Malaysia, has identified handling and disposal of unused medicines as one of the five research domains stipulated in the Pharmacy Research Priorities of Malaysia [20]. Since 2010, Malaysia introduced the Medicine Return Programme (MRP) and developed a national guideline on handling returned medicines to safely dispose of unused medicines in the government healthcare facilities [6]. However, noticeable differences still exist in promoting the programme in various government health facilities [21]. Several Malaysian studies have reported that less than a quarter of the population returned unused medicines to the facilities [21–23]. In comparison, Germany and Sweden have better success, with about 50% of unused medicines being returned to the pharmacy. The success in these countries is attributed to the targeted information campaigns that have increased public awareness regarding the impact of improper disposal of unused medicines [24].

It is desirable to dispose of unused medicine by participating in the MRP [5, 6]. Other ways of disposal by household consumers would be deemed improper. To date, several studies in Malaysia have reported that most people (around 75%) dispose of their unused medicine into household garbage, sink or drainage systems [21–23]. However, we are unaware of any Malaysian study examining the impact of improper disposal of these unused medicines on humans, animals, and aquatic lives.

Several studies in Malaysia examining the extent of unused medicines only focused on patients in government healthcare facilities or universities’ populations [2, 21–23]. Our study covered a broader demographic, which included the general public from the areas surrounding the government and private healthcare facilities, community pharmacies and public places. We aimed to (a) describe the disposal practice of unused medicines and (b) identify the factors associated with participants having unused medicine. The findings would help plan strategies to address the percentage of unused medicines in Malaysia.

## Methods

### Study design

This study was a cross-sectional interview-based survey. We conducted the face to face interviews using a structured questionnaire.

### Research ethics approval

The University of Malaya Research Ethics Committee approved the project (UM.TNC2/UMREC – 1074). All methods were performed in accordance with the ethical guidelines of the Declaration of Helsinki. We obtained written informed consent from all subjects.

### Participants and setting

The study participants consisted of the general public in public places in the locations within the vicinity of eight major government hospitals, one university hospital, three primary government health clinics, six pharmacies in three major districts, three universities, one supermarket, three public parks in two major districts, in the state of Kuala Lumpur (2 million population) and Selangor (6.5 million population) [25]. We excluded those who have not used any medication for the past 6 months, aged below 18 years old, and are unable or refused to participate in the interview.

### Sample size and sampling technique

We used the Raosoft® sample size calculator [26] to calculate the sample size, based on a 95% confidence interval and margin of error of 5% and estimation of 70% participation rate reported in a national survey in Malaysia [27]. Thus, the estimated minimum sample size required was 641 individuals. We used the convenience sampling (non-probability sampling) method to recruit potential participants because of the constraints of recruiting using other probability sampling methods such as stratified or random sampling of participants at public places. Several similar studies have also adopted the convenience sampling technique [22, 28–30].

### Study instrument

The questionnaire was adopted and modified from previously published studies [31, 32]. The questionnaire consisted of three sections (Additional file 1). The first section collects the respondents’ demographic information such as their age, gender, ethnicity, and highest education level. The second section collects health data such as whether the respondents had chronic illnesses (self-reported by participants), pay for their medication (Table 1), and their medication utilisation and disposal pattern. Finally, the third section consisted of questions about the respondents’ knowledge, opinions and practice for unused medicine (Table 1).

**Table 1:** Description of variables used in the questionnaire

Variable	Questions in the questionnaire	Response
Chronic illnesses	Do you have any illnesses that require long-term treatment? (eg. diabetes, hypertension, asthma) “Participants self-reported their illnesses”	Yes/No
Pay for medicine	Do you pay for those medications?	Yes/No
Advised on disposal	Have you ever been advised by a health care professional about proper medicine disposal?	Yes/No
Perceived environmental risk	How do you perceive the risk of unsafe disposal of medicine to the environment?	Low/Medium/High
Awareness of MRP	Are you aware of the medicine return programme? (A medicine return programme is a programme where you voluntarily return your unused medications to a designated facility, such as hospital, pharmacy, or doctor’s clinic)	Yes/No
MRP Participation	Have you ever participated in any medicine return programme by returning your unused medication?	Yes/No
Willing to participate in MRP	Would you be willing to use a medicine return programme if it is available near you, such as at, hospital, pharmacy or doctor’s clinic?	Yes/No

The original questionnaire was written in the English language, and it was then translated into the national language of Malaysia, Bahasa Malaysia. The Bahasa Malaysia translated questionnaire was then back-translated to English to validate the translation. A pilot test was conducted on 50 respondents. It has resulted in some minor change in the questions. The data from the pilot test were not used in the analysis.

### Data collection

A pharmacist (the first author, LSW) and a pharmacy student trained by the pharmacist collected the data from October 2020 to February 2021. The interviewers explained the purpose of the survey, assured the participants of their data anonymity and confidentiality, and recruited only participants who fulfilled the inclusion criteria and consented in writing to participate in the study. The interviewers conducted the interview using the structured questionnaire.

### Data analysis

The cleaned data were entered into Statistical Package for the Social Sciences (SPSS) version 26 (SPSS Inc., Chicago, IL, USA) for the statistical analysis.

Descriptive statistics, such as frequencies and percentage, were used to describe the data. We used the Chi-square test for bivariate analysis to examine the relationships between categorical variables. Statistically significant association in any of the explanatory variables were identified at the level of *p* < 0.05. We included factors found to be significant in the univariate analysis into the multivariate logistic regression model.

### Model building

“Having unused medicine” was the dependent variable and is defined as participant possessing medicines that are no longer consumed by them [1]. Since the dependent variable was a dichotomous variable, we coded 0 = “No” response and 1 = “Yes” response. The independent variables were age in years; gender; ethnicity (Malay, Chinese, Indian and others); Highest education level (None & Primary; Secondary; Tertiary); Having chronic illnesses (self-reported by the participants) (Yes/No); Pay for their medicines (Yes/No); Advice by healthcare professionals about proper disposal (Yes/No); Perceived environmental risk (Low Risk/ Medium Risk/ High Risk); Awareness of the medicine return programme (Yes/No); Participation in the medicine return programme (Yes/No); and, Willingness to participate in a MRP (Yes/No).

We did a univariate logistic regression analysis for the identification of independent variables to be included in the model. We used the significant level at *p* < 0.1 to select variables for the univariate logistic regression analysis because, at a level of *p* < 0.05, variables known to be important would have been excluded [33]. Several other studies [34–36] also used the same level (*p* < 0.01). Non-ordered categorical data with more than two levels (e.g. ethnicity) were entered as *k* - 1 dummy variables. For ethnicity, Malay was made the reference group. As for the ordered category data with more than two levels, i.e. “Education” and “Perceived Environmental Risk”, the variable was entered as *k* - 1 dummy variables. The lowest level (“non and primary level” and “perceived low risk”) were used as the reference groups. As income level was significantly associated with education level, we chose education level as the predictor variable to be included in the logistic regression model to avoid multicollinearity issue [37]. Variables found to be statistically significant from the univariate analysis were simultaneously entered into the multivariate logistic regression model to determine their independent predictive value for the dependent variable.

## Results

### Characteristics of samples

Out of 1230 participants approached, 1184 agreed to participate, giving the study a response rate of 96%. The middle age group (between 50 to 69 years old) made up the highest number (40%, *p* < 0.001) of the sample (Table 2). The gender distribution was almost equal. The Malays ethnic group represents the ethnic distribution of the urban Malaysian population. Most of the respondents had chronic illnesses (65.5%, *p* < 0.001) and paid for their medicines at some points in time (58.6%, p < 0.001) (Table 2). Most respondents reported they did not receive education on the proper disposal of unused medicines (75.7%, p < 0.001). About half of the participants were unaware that they could return unused medicines (*p* < 0.001). Out of those who took part in the programme (20%, p < 0.001), the majority still had unused medicines.

**Table 2:** Demographic and characteristics of the respondents and their association with unused medicines

	Total *N* = 1184 n (% over Total)		Having unused medicines, *N* = 995 (84%) n (%)		*p*
Age					< 0.001
18–29	266	(22.5)	259	(97.4)	
30–39	164	(13.9)	150	(91.5)	
40–49	153	(12.9)	131	(85.6)	
50–59	255	(21.5)	202	(79.2)	
60–69	229	(19.3)	164	(71.6)	
> 70	117	(9.9)	89	(76.1)	
Gender					0.013
Male	585	(49.4)	476	(81.4)	
Female	599	(50.6)	519	(86.6)	
Ethnicity					< 0.001
Malay	646	(54.6)	515	(79.7)	
Chinese	423	(35.7)	385	(91.0)	
Indian	111	(9.4)	92	(82.9)	
Other	4	(0.3)	3	(75.0)	
Highest education level					< 0.001
Primary	136	(11.5)	100	(73.5)	
Secondary	470	(39.7)	379	(80.6)	
Tertiary	578	(48.8)	516	(89.3)	
Chronic illnesses					< 0.001
Yes	775	(65.5)	588	(75.9)	
No	409	(34.5)	407	(99.5)	
Pay for medicines					< 0.001
Yes	694	(58.6)	664	(95.7)	
No	490	(41.4)	331	(67.6)	
Advised on disposal					< 0.001
Yes	288	(24.3)	210	(72.9)	
No	896	(75.7)	785	(87.6)	
Perceived environmental risk					< 0.001
Low	313	(26.4)	264	(84.3)	
Medium	276	(23.3)	251	(90.9)	
High	595	(50.3)	480	(80.7)	
Aware of Medicines Return Programme (MRP)					< 0.001
Yes	575	(48.6)	444	(77.2)	
No	609	(51.4)	551	(90.5)	
MRP participation					< 0.001
Yes	228	(19.3)	167	(73.2)	
No	956	(80.7)	828	(86.6)	
Willing to participate in MRP					< 0.001
Yes	1002	(84.6)	824	(82.2)	
No	182	(15.4)	171	(94.0)	

### Association of participants characteristics with unused medicine

Overall, the percentage of respondents with unused medicines was high (84%). Table 2 shows that as the age group increases, the percentage of unused medications decreases (p < 0.001). Of the four ethnic groups, the Chinese had the highest percentage of unused medicines compared to the Malay, Indian and other ethnicities. The percentage difference between the groups was statistically significant. Respondents with higher education level had a higher percentage of unused medicines. Patients with acute illnesses had a higher percentage of unused medicines than those with chronic illnesses, and the percentage difference was statistically significant.

Table 2 also shows that participants who paid for their medications had a statistically significant higher percentage of unused medicines than non-paying patients. As expected, we found that those respondents who received advice about the proper disposal of unused medications had fewer unused medicines. Likewise, participants who were aware of the MRP and willing to participate had fewer unused medicines.

### Unused medicines

**Fig. 1:**
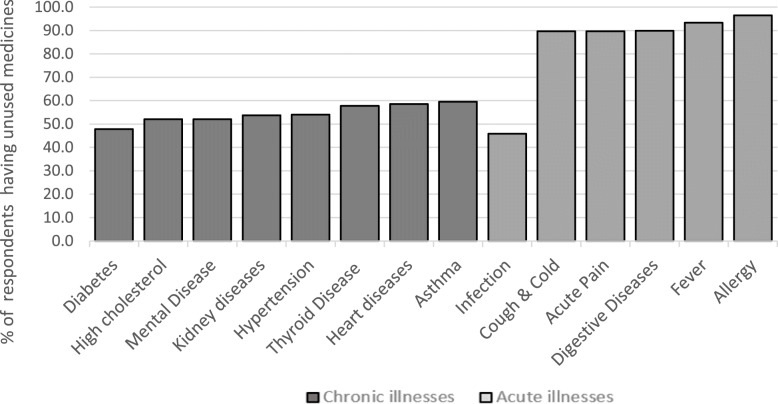
Percentage of respondents with unused medicine for chronic and acute illnesses

We classified diabetes, high cholesterol, mental diseases, kidney diseases, hypertension, thyroid diseases, heart diseases and asthma as chronic illnesses. In contrast, we classified mild infection, cough and cold, acute pain, digestive diseases, fever, and allergy as acute illnesses. Figure 1 shows that 50 to 60% of respondents who used medicines for chronic illnesses had unused medicines. The lowest were respondents with medicines for diabetes, while the highest were respondents with asthma medicines. A high percentage (above 90%) of the respondents who used medicines for acute illnesses had unused medications. The only exception in this category was medicines for infectious diseases.

### Reasons for having unused medicines

Table 3 shows the reasons for having unused medicines. For chronic illnesses, the leading cause of unused medication was non-adherence. Non-adherence included participants who did not take or forgot to take their medicines according to the prescription. Meanwhile, for respondents with acute illnesses, the main reason for having unused medications was because their medical conditions have improved, and they did not need all the amount prescribed.

**Table 3:** Reasons for having unused medicines

	Chronic illnesses n (%)		Acute illness n (%)	
Did not take according to the prescription/forgot	570	(68.5)	69	(5.8)
The doctor has changed the prescription or dosage	128	(15.4)	5	(0.4)
Did not need all that was prescribed/too many	52	(6.3)	128	(10.8)
Had a bad reaction or side effect	21	(2.5)	13	(1.1)
Illness condition improved	22	(2.6)	943	(79.2)
Medicine has expired	9	(1.1)	24	(2.0)
Patient deceased	6	(0.7)	0	(0.0)
Getting supply from multiple centres	5	(0.6)	2	(0.2)
Other reasons	19	(2.3)	6	(0.5)

### Methods of disposing of unused medicines

Table 4 shows the disposal methods of unused medicines depended on the category of medicines (chronic or acute illnesses). The percentage of respondents who kept their unused medicines for future use was high for both categories, with a higher percentage for respondents with acute illnesses. Nearly one-third of those with chronic illnesses returned the unused medicines to the MRP facilities, compared to a very low percentage (1.5%) for those with acute illnesses. The rest of the respondents kept the unused medicines in the cabinet, threw the unused medicines in the trash, flush the medicines in the toilet or drain or gave them to someone else.

**Table 4:** Methods of disposal

	Chronic illness n (%)		Acute illnesses n (%)	
Kept it for future use	257	(30.9)	547	(46.2)
Give them to Medicine-Return-Programme (MRP) facility	224	(26.9)	18	(1.5)
Kept it in a cabinet (still have it)	182	(21.9)	290	(24.4)
Threw it in the trash/garbage bin	149	(17.9)	286	(24.0)
Flushed it down the toilet/drain	8	(1.0)	14	(1.2)
Gave it to someone who would use it	5	(0.6)	31	(2.6)
Other methods	7	(0.8)	1	(0.1)

### Predictor of ‘having unused medicines’

In the preliminary univariate logistic regression analysis, nine predictor variables (Table 5) were significantly related to the dependent variable, ‘Having unused medicine’, at the level of *p* = 0.1 [33]. We included all these significant predictor variables for the subsequent multivariate logistic regression analysis. The multivariate logistic regression model predicted the likelihood of variables associated with ‘Having unused medicines” at the significant level of *p* < 0.05.

**Table 5:** Predictor of “having unused medicines” in multivariate analysis

Variables^a^	B	S.E.	Significance	Odds Ratio	95% C.I.	
					Lower	Upper
Age	− 0.005	0.007	0.525	0.995	0.981	1.010
Ethinicity			0.939			
Chinese	−0.108	0.237	0.648	0.897	0.564	1.428
Indian	−0.110	0.310	0.722	0.896	0.488	1.645
Other	−0.514	1.409	0.715	0.598	0.038	9.466
Gender	−0.078	0.187	0.675	0.925	0.641	1.333
Education			0.919			
Secondary	0.031	0.260	0.906	1.031	0.619	1.718
Tertiary	−0.055	0.288	0.849	0.947	0.539	1.664
Acute Illnesses	3.396	0.737	0.000	29.844	7.037	126.559
Pay for Medicines	1.794	0.233	0.000	6.012	3.811	9.485
Advised Disposal	−0.292	0.191	0.127	0.747	0.514	1.086
Perceived Environmental Risk			0.092			
Medium	0.386	0.291	0.185	1.472	0.832	2.604
High	−0.180	0.216	0.405	0.836	0.548	1.275
Willingness to participate in MRP	−0.923	0.353	0.009	2.517	1.260	5.028
Constant	0.927	0.566	2.685	2.527		

^a.^ The reference categories for the variables are as follows: Race: Malay; Gender: Male; Pay for medicine: no; Education: None & Primary; Acute illnesses: no; Advised on Disposal: no; Perceived Environmental Risk: Low; Unwilling to participate in MRP: Yes

The multivariate logistic regression model explained 20.7% (Cox and Snell R^2^), and 35.3% (Nagelkerke R^2^) of the variance of confidence, and the overall percentage of cases correctly predicted was 84%. Three independent variables showed statistical significance in the multivariate logistic regression model (Table 5) [38]. The strongest variable that predicted whether a person has unused medicines were medicines used for acute illnesses. The odds of having unused medicines for a person using medicines for acute illnesses were 29.8 (*p* < 0.001) times higher than those using medicines for chronic illnesses. The other significant variable was ‘pay for medicine’. The odds of having unused medicines for respondents who paid for their medicines were 6.0 (*P* < 0.001) times higher than non-payers. The variable “willingness to participate in MRP” was also statistically significant. The odds of having unused medicines for respondents who were willing to participate in the MRP were 2.5 (*p* = 0.009) times lower than that of those who were unwilling to participate.

## Discussion

This study aimed to describe the disposal practice of unused medicines and to identify factors associated with participants having unused medicines. As we predicted, the behaviour of the patients consuming their medications differs between medicines used for chronic illnesses and acute illnesses. Even though we did not explore prescription behaviour, this difference could also indicate that for acute illnesses, patients receive more than what they needed [32].

However, the primary reason for having unused medicines for patients with chronic illnesses was non-adherence. Over two-thirds of these respondents reported they forgot to take medicines or did not take them according to the prescription. One solution to resolve the non-adherence issue is by conducting effective medication review and counselling [39, 40].

Meanwhile, respondents with acute illnesses had unused medicines because their conditions improved or received more than what they needed. Oversupply of medicines could be due to prescribing and dispensing behaviour [41]. Therefore, it is crucial to reconsider the duration of medicine supply for acute illnesses.

Unused medicines are often kept for future use. Stored medicines would end up forgotten, expired and subsequently discarded in the trash. Efforts are needed to educate patients to return unused medicines to MRP facilities. The lack of proper advice and understanding about self-medication could lead to abuse, poisoning, and unintended medication use by others [11, 12].

The multivariate logistic regression analysis revealed three variables to be statistically significant predictors for respondents having unused medicines. The strongest variable that predicts whether a person has unused medicine is a person using medicines for acute illnesses such as cough and cold, diarrhoea, acute pain and fever. The odds of having unused medicines for respondents with acute illnesses were 29.8 (*P* < 0001) times higher compared to those using medicines for chronic illnesses such as hypertension, diabetes, high cholesterol and asthma. Our finding is consistent with several previous studies. For example, an Eastern Ethiopian study reported that medication used for acute illnesses comprises 90% of unused medicines [19]. Another study from India [42] reported 83%, while a Nigerian study [43] reported a figure of 53%. Treatment for acute illnesses is short-termed, and we would expect participants to stop taking their medicines if their illness or condition improved. Our study also shows the majority (80%) of our respondents with acute illnesses stopped taking their medicines because their conditions improved. A similar figure. (81%) is seen from a Ghanian study [44]. However, the figures are low for developed countries like the USA [45] and New Zealand [14]. A large percentage of unused medicines for acute illness in less developed countries highlighted the possibility that respondents received an oversupply of medicines for their conditions and have kept their medication for future use [14]. These medicines would eventually be expired and improperly discarded. Healthcare professionals, particularly pharmacists, should optimise the amount supplied and practice rational dispensing to address this issue.

Malaysia has a dual-tiered public-private healthcare services system [46]. In the public healthcare sectors (government hospitals and clinics), patients do not pay for their medicines. In contrast, patients pay for their medicines in the private healthcare sector which consists of private hospitals, clinics and community pharmacies. Thus, a person has a choice to obtain their medicines from either the public healthcare sectors for free or pay from the private healthcare sector. We found that the predictor variable “pay for medicines” is significantly associated with having unused medicines. The odds of having unused medicines for respondents who paid for their medicines were 6.0 times (*p* < 0.001) higher than non-payer. This can be explained by the fact that the private healthcare sector does not implement the MRP in Malaysia, unlike the public healthcare sector. Therefore, patients who pay for their medicines lack awareness about MRP and do not have available MRP facilities to return their unused medicines. Thus, it is logical to extend the MRP and related patient education to private healthcare facilities to address the improper disposal of unused medicine.

The third predictor variable is “willingness to participate in an MRP”. The odds of having unused medicines for a respondent who is willing to participate in the MRP are 2.5 times (*p* = 0.009) lower than those who are unwilling to participate. We postulate that respondents who are willing to return their unused medicine would be more aware of the importance of the MRP programme.

We are surprised to find that healthcare professional’s advice on proper disposal was not a significant predictor for having unused medicines. More work needs to be done to increase awareness about the importance of participating in the MRP. We would advocate increasing participation of MRP by having easily accessible collection facilities. The MRP should also be extended to private healthcare facilities, such as community pharmacies, private clinics and hospitals, as suggested by Persson [24].

### Strengths and limitations

Our study’s strengths are the substantial sample size (*n* = 1184) and the high respondence rate of 96%; thus data generated are reliable. In addition, the survey has included a broader demographic area than the previous studies in Malaysia [2, 21–23]. Finally, we used a multivariate logistic regression model to predict the likelihood of having unused medicines. The overall percentage of cases correctly predicted was high (84%).

However, the study had several limitations. First, we conducted the study on a population sample from Kuala Lumpur and Selangor, most of whom are urban and suburban. Therefore, we cannot generalise the finding to the entire Malaysian population that also comprises the rural community. Second, the convenience sampling method has the limitation of variability, and we cannot control and measure the possible bias. Furthermore, we cannot generalise the results from the collected data beyond the sample [47]. Third, the survey questionnaire depends on the respondents’ memories and probably suffers from social acceptability bias [27].

To overcome the limitation, one could expand the future study to cover the rural population. In addition, interviews conducted in the respondents’ home could allow the researcher to determine the actual type and amount of the unused medicines, thus addressing recall bias and social acceptability bias. Finally, the home visit may also identify the respondents with a better sampling method and is suitable for future follow-up surveys and measurements of improvement in the proper medication used and disposal behaviours.

## Conclusion

The prevalence of unused medicines and their improper disposal was high in Malaysia. Important predictor variables, which predict having unused medicines, are related to whether a person uses medicines for chronic or acute illnesses, paid for the medicines, and willingness to participate in the MRP. Rationale prescription practice with optimal dispensing quantity and broader coverage of MRP facilities could reduce unused medicine and encourage proper disposal. The findings would be useful for planning strategies to minimise unused medicines and foster the appropriate disposal practice.

## Supplementary Information

**Additional file 1: **Unused Medicines**.** Survey questionnaire.

## Data Availability

The analysis results generated in the study are presented in this published article. The research data set are available upon request from the corresponding author.

## Abbreviations

SPSS: Statistical Package for the Social Sciences
MRP: Medicine Return Programme
OR: Odds Ratio
MOH: Ministry of Health, Malaysia
UMREC: Universiti Malaya Research Ethics Committee.
