# Supplementary material for: Disposal practice and factors associated with unused medicines in Malaysia: a cross-sectional study
Source: BMC Public Health. 2021 Sep 16;21:1695. doi: 10.1186/s12889-021-11676-x (PMC8447783; doi:10.1186/s12889-021-11676-x)
Supplement: Supplementary file 1 — Additional file 1: Unused Medicines. Survey questionnaire. [file 12889_2021_11676_MOESM1_ESM.pdf]

Survey: Unused Medicines

Location:

Date:

We are conducting a survey to learn more about unused medicines.
The information obtained from this survey would be able to help us to recommend ways to prevent medication wastage and its safe disposal.
Please be sure that all responses will be anonymous. This survey should only take about 5-10 minutes.
Thank you for your consent to participate in this survey. Let's start now.

About yourself

1. Your age ______ years old
2. Your race ______
3. Your gender ☐ 1. Male ☐ 2. Female
4. Your highest education level: ______
5. Your monthly income range: (indicate an approximate value)
   - ☐ 1. <RM1,000
   - ☐ 2. RM1,001 – 3,000
   - ☐ 3. RM3,001 – 5,000
   - ☐ 4. RM5,001 – 10,000
   - ☐ 5. >RM10,000
   - ☐ 0. No income

About your medication

6. Do you use any medication for the past six (6) months
   - ☐ 2. Yes ☐ 1. No
7. Do you have any illnesses that require long-term treatment? (eg. diabetes, hypertension, asthma etc.)
   - ☐ 2. Yes ☐ 1. No
8. Where do you get the supply of medication? (you may choose more than one answer)
   - ☐ Government hospital or clinic
   - ☐ Private hospital or clinic
   - ☐ Retail/community pharmacy (e.g. Guardian or Caring Pharmacy etc.)
9. Do you pay for those medications?
   - ☐ 0. Yes
   - ☐ 0. No
   - ☐ 1. Sometimes (specify) ______

Survey: Unused Medicines

10. Please provide the details of medicines you have at home (for the period of last six months):

**Type of medicine for/illness**? (or medicine name)	How many **days of supply** do you normally get?	How many **days left unused** (estimate)?	What are the **reasons for not taking/finishing** the medication? §	How did you **dispose of** the unused medicines? **
A. Hypertension				
B. Diabetes				
C. High cholesterol				
D. Heart diseases				
E. Kidney diseases				
F. Pain (muscle/gout/ headache/toothache)				
G. Infection (antibiotic)				
H. Digestive disorder				
J. Depression				
K. Cough & cold				
L. Allergy				
M. Fever				
N. Others (specify)				
O. Asthma				
P. Mental Disease				

Examples:

§ The **reasons for not taking/finishing** the medication?

1. Prescription or dosage was changed by a doctor
2. Did not take according to the prescription/forgot
3. Did not need all that was prescribed/too many
4. Had a bad reaction or side effect
5. Illness condition improved
6. Medicine has expired
7. Medicine turn bad (smell/taste/look)
8. It was lost or misplaced
9. It was stolen
10. Patient deceased
11. Getting supply from multiple centres
12. Other reasons (specify)

How did you **dispose of the unused medications?

1. Threw it in the trash/garbage bin
2. Flushed it down the toilet/drain
3. Look it to a "Medicine Take-Back" facility
4. Gave it to someone who would use it
5. Kept it in a cabinet (still have it)
6. Kept it for future use
7. Other Methods (specify)

Survey: Unused Medicines

About medication disposal and Take-back Programme

11. Have you ever been advised by a health care professional about proper medicine disposal?

- ☐ 1. Yes
- ☐ 0. No

12. How do you perceive the risk of unsafe disposal of medicine to the environment?
Please rate the risk as follow (1 being low risk, 5 being very high risk)

☐ 1. Low Risk ☐ 2. Medium Risk ☐ 3. High Risk

13. Are you aware of medicine return programme?
(A medicine return programme is a programme where you voluntarily return your unused medications to a designated facility, such as hospital, pharmacy, or doctor's clinic)

- ☐ 1. Yes
- ☐ 0. No (Go to Q16)

14. Have you ever participated in any medicine return programme by returning your unused medication?

- ☐ 1. Yes (Go to Q16)
- ☐ 0. No

15. Could you please explain or tell me why you have not participated in a medicine return programme?

- ☐ 1. It is inconvenient
- ☐ 2. Too far to travel
- ☐ 3. No time
- ☐ 4. Not concerned about unused medication disposal
- ☐ 5. Keep medications for future use
- ☐ 6. Concern that the prescriber/pharmacist will blame me
- ☐ 7. Some other reasons (specify):

16. Would you be willing to use a medicine return programme if it is available near you, such as at, hospital, pharmacy or doctor's clinic?

- ☐ 1. Yes
- ☐ 0. No

END

That is the end of the survey. Thank you for your time today. If you have any questions about how to properly dispose of unused medications, please ask your healthcare professionals at your next visit. Have a good day.
